# Reliability assessments of an islanded hybrid PV-diesel-battery system for a typical rural community in Nigeria

**DOI:** 10.1016/j.heliyon.2019.e01632

**Published:** 2019-05-10

**Authors:** Ayodele Benjamin Esan, Ayoade Felix Agbetuyi, Oghenevogaga Oghorada, Kingsley Ogbeide, Ayokunle. A. Awelewa, A. Esan Afolabi

**Affiliations:** a Department of Electrical & Information Engineering, Landmark University, Omu-Aran, Nigeria; b Department of Electrical & Information Engineering, Covenant University, Ota, Nigeria

**Keywords:** Energy, Electrical engineering

## Abstract

This paper presents the use of a novel approach in assessing the generation reliability of a hybrid mini-grid system (HMS) based on the optimal design result obtained from the HOMER software. A typical Nigerian rural community – Lade II in Kwara State was used as a case study where the energy demand for the residential and commercial loads was 2.5MWh/day and 171kWh/day respectively. The optimized HMS results from HOMER comprising of a solar photovoltaic (PV) array (1.5MW), diesel generators (350kW) and battery storage (1200 units) has a combined least net present cost of $4,909,206 and a levelized electricity tariff of $0.396 per kWh. Contrasting the HMS with a diesel-only system for the community, an approximate 97% reduction in all pollutant emissions was observed. Furthermore, fluctuations in diesel fuel prices, variations in average solar insolation, and variations in the solar PV's capital/replacement costs were utilized in conducting a sensitivity analysis for the HMS. The capacity outage probability table (COPT) was utilized in validating the reliability of the simulation results obtained from HOMER. The HMS was observed to experience a load loss of 0.769MW, 0.594MW & 0.419MW when zero, one and two diesel generator(s) respectively were operational for all of the Solar PV's and Batteries being off-line. The loss of load probability (LOLP), loss of load expectation (LOLE), and total expected load loss (ELL) obtained from the COPT were 5.76 × 10^−8^, 5.0457 × 10^−4^ hr/yr and 0.025344Watt respectively. The results show the reliability of the HMS and also depicts a highly economical and feasible hybrid energy system.

## 1 Introduction

Inadequate power supply has been the bane of Nigeria's industrial and economic development resulting in the wastage of perishable farm produce, the underperformance of agro-allied industries, reduced efficiency in manufacturing and service industries. These economic losses has increased the poverty level among her citizens [1]. About 50% of the populace are said to be living without electricity with majority residing in the rural areas. Despite policies enacted by many of the Sub-Saharan Africa & South Asian countries, approximately 2 billion people still lack access to electricity in these areas [2]. To meet their basic electricity needs, many households and commercial centers rely heavily on fossil-based secondary energy sources such as dual-purpose kerosene (DPK), premium motor spirit (PMS), diesel generators etc. which are costly to operate and are not pollutant free [3, 4, 5].

The economic competitiveness of small-scale solar PV systems, wind generators, small hydro systems has well been established. The government now makes policies in support of the development of off-grid energy sources for improved electricity delivery especially to rural communities [6]. The Rural Electrification Agency (REA) saddled with the responsibility of rural electrification in Nigeria has identified Nigeria as the biggest and most attractive off-grid opportunity in Africa because of the huge energy needs of the large populace. The REA has established the rural electrification fund (REF) as a strategy to realize the off-grid electrification plans [7]. The REF receives funding from the World Bank and the Federal Government of Nigeria (FGN). The World Bank in collaboration with the FGN have created a five-year plan for the national electrification project (NEP) set out by Nigeria [7] with a funding sum of 150 million United States Dollars (USD). According to the REA, about 1200 mini-grids would be developed to serve 200,000 households and 50,000 local enterprises. To this end, the REA conducted some feasibility studies covering over 200 locations across the country to ascertain the off-grid energy potentials available in each location and to determine which renewable energy sources is more suitable for each location. The study showed that solar energy will provide the best forms of renewable energy technology that could be harnessed through the use of solar photovoltaic (SPV's) technologies, concentrated solar power (CSP) or solar home systems (SHSs) in many of the locations especially in the northern part of the country [7].

Due to the stochastic nature and seasonal variations of sunshine hours and wind speed, using a single renewable energy source may not be the best option for a community in terms of reliability. For this reason, many energy system designers consider a hybrid energy system where two or more renewable energy sources are combined alongside battery storage systems and sometimes diesel generator(s) as backup [8]. Some hybrid combinations include solar PV/wind/battery systems, solar PV/battery systems, solar PV/wind/diesel systems etc. This sort of energy designs increases the reliability of the hybrid system exponentially with reliability indices such as the loss of load probability (LOLP), loss of load expectation (LOLE) and expected load loss (ELL) being minimal. Previous works on hybrid mini/microgrid designs and techno-economic analysis have adopted a wide variety of artificial intelligence algorithms, analytical approaches and even the use of software such as the Hybrid Optimization Model for Electric Renewables (HOMER) which was developed by the National Renewable Energy Laboratory.

The authors in [9, 10, 11, 12, 13, 14, 15, 16, 17, 18, 19, 20] used the HOMER software for hybrid energy designs and techno-economic analysis. In [9], a system model and performance evaluation were conducted on two decentralized power stations in Sabah Malaysia where each station contained different combinations of solar PV's, diesel generator, storage batteries, and system converters. In [10], the economic analysis of utilizing a hybrid energy system – solar PV/wind and diesel generator was conducted for remote areas of Southern Ghana. The optimal system obtained comprised of an 80kW solar PV array, a 100kW wind turbine, a 100kW diesel generator, a 60kW converter and 60 units of batteries having $0.281/kWh as the least cost of electricity (COE). In [11], four different scenarios were considered for an Integrated Renewable Energy System for seven un-electrified villages in Almara District of Uttarakhand State, India where different combinations of micro hydropower, biomass, biogas, solar PV and wind energy were utilized. In [12], three energy sources was considered namely solar, wind and diesel energy sources for off-grid applications. Sequel to the techno-economic analysis, the authors suggested the use of diesel generators for basic level energy demands and a PV-diesel hybrid for higher energy demands. In [13], a normalized performance index and techno-economic analysis for a solar PV plant in an Indian isolated island of Andama and Nicobar was conducted. The result showed an optimal configuration comprising a 2.5 kW PV array, 12 numbers of battery and a converter size of 2kW. The cost of electricity was also found to be $0.398/kWh with a Net Present Cost (NPC) of $9,637 and an Operating Cost (OC) of $224/year. The authors in [14] using load data obtained from an electric machinery laboratory in Kavale town, Greece examined the use of hydrogen technologies as part of the energy mix with the solar PVs and batteries for the design of a hybrid energy system. The net present costs for different combinations of PV, hydrogen generators and storage batteries were determined. In [15], the authors performed the techno-economic feasibility of 3 energy sources comprising of solar, wind & diesel energy sources in selected villages across all the six geo-political zones in Nigeria. The result showed that the PV/diesel/battery system had the least NPC among other configurations studied for all six sites considered. Similarly, in [16], the techno-economic analysis of utilizing a hybrid energy system in supplying electricity to typical rural healthcare (RHC) facilities in selected locations across the six geo-political zones in Nigeria was assessed. The author's results revealed the hybrid system of solar PV-battery-wind-diesel as being the optimum for RHC applications in Sokoto, Maiduguri, Jos and Enugu, and solar PV-battery-diesel as being the optimum for RHC applications within Iseyin and Portharcourt. In [17], the authors considered the use of solar and wind energy for a hybrid system in energizing a remote mobile base station transceiver station in Nigeria. In the optimization results, two best system configurations were realized i.e. PV/diesel/battery system and PV/wind/diesel/battery system. However, when compared with the conventional standalone diesel generator, the solar PV/diesel/battery system proved most economically viable with the least cost of electricity as $0.409/kWh and a net present cost of $69,811. Also, a commensurate reduction in CO_2_ by about 16.4 tons/year was realized compared to using the diesel generator alone. In [18], the authors assessed the techno-economic impact of a grid-tied solar PV/wind energy system for a cattle farm in an Algerian desert considering three different scenarios with a yearly energy consumption of 6.71kWh and a peak load demand of 7.7kW. Using HOMER, It was found that the optimized solutions from the 3 scenarios based on the net present cost satisfied the daily farm energy consumption of 18kWh. In [19], for an urban area in Cape Town, South Africa, HOMER was used in performing the techno-economic feasibility of a rooftop solar PV, battery storages and diesel generator in sync with the evaluation of a grid-tied mode as a back-up strategy. The result showed an optimal combination comprising 129kW of solar PV, 126 strings of batteries, 140kW diesel generator and 60.9kW converter with a Levelized Cost of Electricity (LCOE) of 0.509$/kWh and a net present cost of $1.64 million. In [20], the authors performed a poly-generative combination of an islanded hybrid renewable energy system for a large resort center situated in the South China Sea, Malaysia. Using HOMER for their analysis, their results comprised a solar PV/wind/diesel generator/converter and battery with a net present cost of $17.15 million, cost of electricity being $0.279 per kWh and a renewable fraction of 41.6%. Compared to the diesel-only system, there were significant differences in the NPC and COE with the CO_2_ level being 5,432,244 kg/year while that of the hybrid system was 2,571,131 kg/year.

The authors in [21, 22, 23, 24, 25, 26, 27, 28, 29, 30, 31] adopted the use of Artificial Intelligence techniques in the design of off-grid hybrid systems.

In [21], a self-made simulation tool developed using Matlab/Simulink was used to model an islanded hybrid power plant comprising of a solar PV array, battery storage, a unitized regenerative fuel cell as the main backup and a diesel generator as a secondary backup system for a strip mall under eight distinct climatic zones in the United States. The result showed that even in optimal or best conditions in terms of the daily solar insolation and temperature, the LCOE of the hybrid system was too costly and for its implementation, a capital reduction above 60% in form of incentives should be given. In [22], the authors introduced a new methodology in the hybrid system design by using small split-diesel generators instead of a single big diesel generator. Using this method, a tri-objective design of a non-grid tied PV-wind-split-diesel/battery poly-generation system for a residential building was performed using the Genetic Algorithm. The results obtained from five different scenarios showed the PV/wind/split-diesel and battery system as the most feasible with a commensurate reduction of 28%, 94%, 46% and 82% in the values of the cost of electricity, dump-energy, Life Cycle Cost (LCC) and CO_2_ emissions respectively when compared with a single big diesel generator. The authors in [23] adopted a novel approach called the preference-inspired co-evolutionary algorithm (PICEA) in the design of a hybrid energy system having three objectives. These tri-objectives were the simultaneous minimizations of the annualized system cost, the loss of power supply probability (LPSP) and fuel emissions. In [24], the authors utilized on two different scales, a hybrid genetic algorithm with particle swarm optimization (GA-PSO) and a multi-objective PSO (MOPSO). Using the GA-PSO and MOPSO, the major aim was to both minimize the total net present cost, initial cost, replacement cost, operations and maintenance costs. The results, however, revealed the PV/Wind turbine and battery system as having the lowest cost with the cost of electricity being 0.508$/kWh. In [25], the authors assessed the technical, economic and environmental performance of a small-scale micro-grid in 3 US Cities comprising of seven system components namely PV's/Wind turbines/lead acid batteries/bio-diesel generators/fuel cells/electrolyzers and H_2_ tanks to provide constant power. An exhaustive search technique was used to locate the system configuration with the least cost of electricity. The result showed a LCOE in the range of $0.32 to $0.42 per kWh with a CO_2_ metric of approximately 1/10^th^ that of an equivalent conventional electric grid for 10 to 50 homes. In [26], a discrete version of the harmony search algorithm (DHS) was used to determine the optimal sizing of a hybrid solar PV/wind/diesel/battery system. The results obtained were thereafter compared with that obtained using the discrete simulated annealing (DSA) algorithm. The final results showed that the DHS algorithm produced a more accurate result compared to the DSA algorithm and yielded an optimal configuration comprising wind/diesel/battery system for the case study. The authors in [27] studied four remote locations in India to determine the optimal sizing and tilting of a hybrid photovoltaic/battery/diesel system. Adaptive Neuro-Fuzzy Inference System (ANFIS) and the Artificial Neural Network (ANN) was used in determining the optimal size of a hybrid system where only the latitude, longitude and altitude for any of the remote locations they studied were used. Using the artificial intelligence techniques, the life cycle cost of the optimized hybrid system with the solar PV only & diesel only generator system were compared to prove the cost-effectiveness of the hybrid system. In [28], the design and techno-economic analysis of a hybrid system – solar PV/battery/diesel generators for a typical Malaysian village household was carried out. Using the Genetic Algorithm, the authors developed algorithm for optimum tilt angle for solar PV panels. Authors in [29] using a probabilistic approach proposed a new reliability indicator called MaxENS which depends on the minimum electric power obtained from the wind and solar radiation per hour and measures the maximum expected energy which the solar and wind sources cannot supply in a period t for Barranquilla city in Colombia. The results from the work when compared with results from the HOMER software, showed an improvement in system reliability since renewable resources use was maximized. In [30], the authors proposed a novel decision-making methodology called the entropy weight which identifies the best configuration of hybrid energy system from a list of probable combinations obtained from HOMER for Yongxing Island in China. The entropy weight method employed a multi-objective function comprising of four crucial indices. In the work, the reliability index comprises of the loss of power supply probability (LPSP) and loss of load expectation (LOLE). In [31], the authors designed a hybrid solar-wind generation microgrid with hydrogen energy storage using a multi-objective particle swarm optimization algorithm where three objective functions; loss of load expected, loss of energy expected, and the annualized cost of the system were minimized.

The authors in [32, 33, 34, 35, 36, 37] focused on the use of analytical techniques for the design and techno-economic analysis of stand-alone and hybrid off-grid energy systems. In [32], the standalone hybrid energy system – solar PVs, wind turbines and diesel generators were analyzed for off-grid application in rural villages in Columbia which had different climatic zones. In [33], different energy configurations were evaluated for the design of a nanogrid for five neighboring houses in Gwagwalada – Abuja in Nigeria. The authors result indicated a 99.2–99.6% availability when a 5–14.5kW solar nanogrid was utilized and a 100% availability when a hybrid option which included a diesel nanogrid was utilized. In [34], a hybrid energy design for a summer house located in Kilis, Turkey was carried out. The authors result showed that a 3kW solar PV, 1kW diesel generator, 2kW converter and 6 units of batteries as the optimal system configuration. To create income generation opportunities in a rural village in Kenya, the authors in [35] designed a solar energy center to provide basic lighting services and mobile phone charging sources. In [36], for remote areas in Tamil Nadu having different climatic zones, the authors analyzed the economic feasibility of installing and operating hybrid systems. It was found that the interior climatic zone had an optimal configuration comprising a solar PV/diesel system. In [37], a bi-objective design model was proposed for a hybrid off-grid PV-BES-Diesel generator system with the aim of identifying the PV plants rated power, battery storage capacity and identifying the technical configurations for each able to jointly reduce the LCOE and the carbon footprint of energy (CFOE).

In describing distribution systems reliability using analytical approaches, the authors in [38] investigated the impact of integrating renewable energy technologies (RET) on the reliability of distribution power systems with the focus primarily on distribution systems. Using a Roy Billinton Test System (not a real case scenario), four distinct case studies were developed, where the first case study solely comprised of a conventional power system with diesel generators. The remaining case studies comprises of a mix of either of the conventional sources and/or renewable energy technologies. The results revealed that for all the remaining 3 case studies, the value of the Expected Energy Not Served (EENS) and the Expected Interruption Cost (ECOST) were far less compared to the first case study. Similar to the work in [38], the authors in [39] utilized the Markov model which assessed the variable characteristics of the system components of a hybrid distributed generation and conducted a thorough reliability assessment on the distribution system which satisfied the consumers load demands and had a high penetration of solar PV's, wind turbine generators and electric storage systems.

Nonetheless, in describing the features of existing reliability models of wind power, the authors in [40] reviewed some reliability assessment algorithms and their commensurate application in wind power related problems. In power system planning, Capacity Outage Probability Table (COPT) as a model was identified amongst other widely adopted power system planning models as a tool to be used to validate generation reliability using indicators such as the loss of load probability (LOLP) and the loss of load expectation (LOLE).

In describing generation system reliability, authors in [41] using the loss of load expectation (LOLE) reliability index analyzed the resultant power system reliability for an already existing power grid when a nuclear power plant was replaced by wind powered plants. In the work, a 696MW capacity nuclear power plant was considered for replacement by three wind power plants having a combined generating capacity of 3,480MW. The first case scenario result made use of real wind data collected over a year and the result showed a reduced system reliability compared with the initial model using the nuclear power plant. Due to the stochastic nature of wind, during less windy period, a maximum LOLE of 2,429 hr./year was experienced but at windy periods, the LOLE obtained was very small and even negligible. Similarly, authors in [42] developed an approach which uses the LOLE computation as a criterion to estimate the needed supplementary operational reserve for the day-ahead operation of an already existing power grid when variable sizes of wind powered generators were installed in the system. From the results, it is observed that with an increased wind power generation uncertainty, the hourly and daily LOLE index increases correspondingly. When the wind generation range was between [-20%, 20%], the daily LOLE observed was 0.0751 hr./day and when the range was between [-80%, 80%], the daily LOLE observed was 0.0808 hr./day.

A research work similar in objectives to that obtained in this paper was performed by authors in [43] where the reliability, economic and environmental benefits of renewable energy sources in a microgrid system was evaluated. The hybrid system comprised a solar photovoltaic array, wind turbine generator, electric storage systems, and a diesel generator. In minimizing the cost of energy, lifecycle cost, emissions cost, the annual cost of load loss and improving the overall benefits of renewable energy technologies in the microgrid, the Fmincon optimization tool in Matlab were utilized using six different case studies. By utilizing basic probability concepts, the reliability performance indicators of their proposed hybrid design such as the expected energy not served (EENS), loss of load expectation (LOLE) and loss of load probability (LOLP) was obtained. The results for all 6 case studies revealed that the integration of renewable energy distributed technologies brought a substantial improvement in the system reliability. Particularly, with the 6^th^ case study which comprised more units of solar PV, wind turbine generators and energy storage systems with reduced power outputs of the diesel generators, the LOLP and LOLE was 2.81 × 10^−10^ and 2.46 × 10^−6^ hr./year respectively.

Almost all reviewed literature in this article had their focus primarily on minimizing one or more forms of economic costs as their objective functions thus generating optimal combinations of renewable energy resources (RERs) with/without conventional sources using software, analytical techniques or multi-objective optimization artificial intelligence algorithms. However as pointed out by authors in [43], to utilize RERs in a microgrid economically and efficiently, the objective functions of the microgrid should not be limited to just economic metrics alone but should also consider some reliability indicators such as the loss of load probability, loss of load expectation, interruption costs, expected energy not served etc. Since cost alone does not determine hybrid system reliability, the need to ascertain or validate other reliability metrics of an optimized hybrid mini-grid system becomes paramount.

In this paper, the Capacity Outage Probability Table (COPT) model is utilized as a novel approach in assessing the reliability of a hybrid mini-grid designed for a typical rural community in Nigeria – Lade II in Kwara State. Using the COPT, the optimal hybrid energy solution (HES) as obtained from the HOMER analysis is used to evaluate reliability indicators such as the loss of load probability (LOLP), loss of load expectation (LOLE) and total expected load loss (ELL) when a capacity outage occurs because of a portion or all the generating sources being off-line. With this reliability indicators, the HES results as obtained from HOMER is thus validated in quantitative terms.

## 2 Methodology

### 2.1 Site description and load assessment

**Fig. 1:**
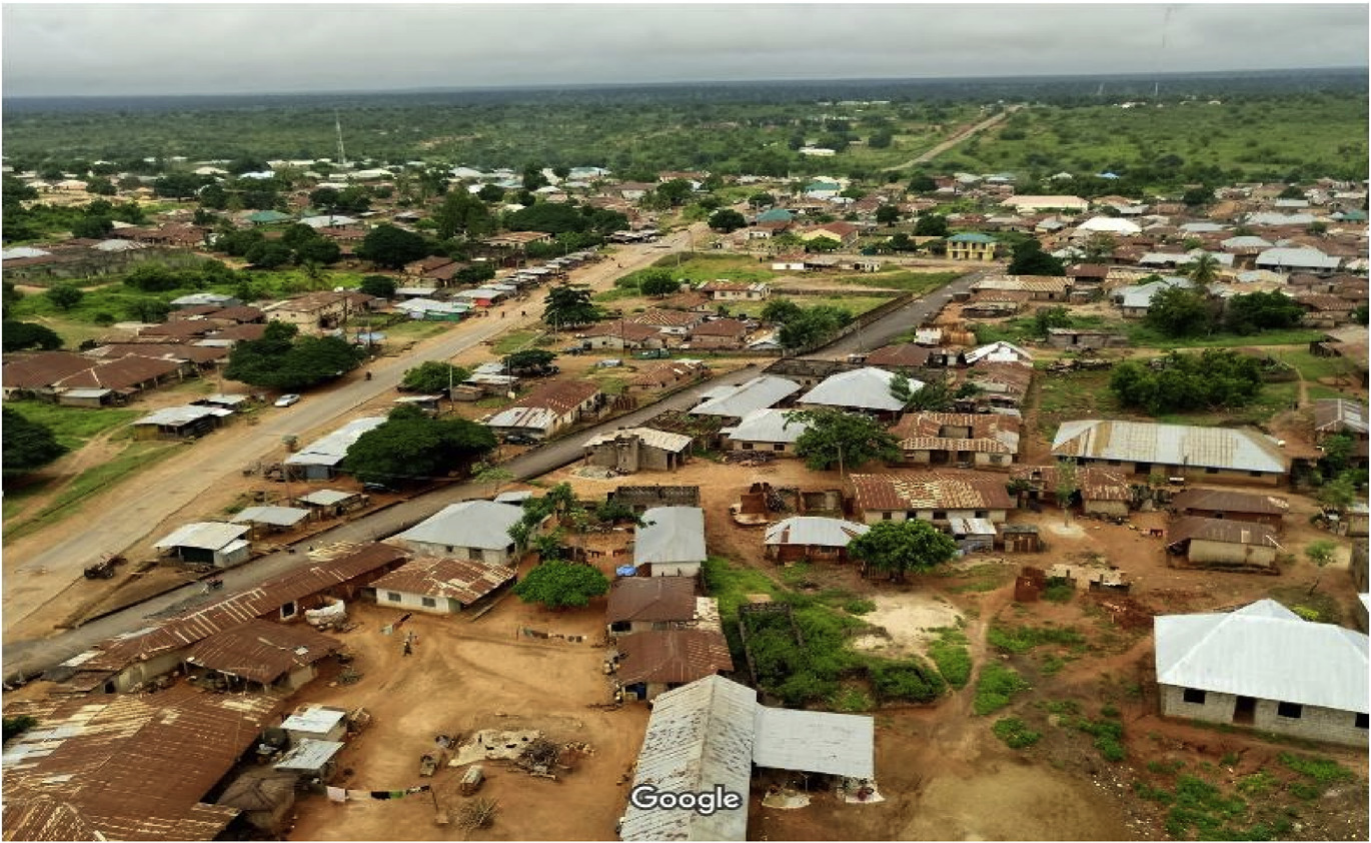
Aerial view of Lade II, Pategi [45].

The location where any proposed hybrid mini-grid design is to be implemented is an essential determinant of the kind of Renewable Energy Technologies (RET) to be deployed. Lade II is a village in Pategi Local Government Area of Kwara state with the geographical coordinates 8.59^o^N latitude and 5.53^o^E longitude. The inhabitants of Pategi are majorly farmers; cultivating and harvesting crops such as melon, millet, cassava, guinea corn. They make lots of local snacks such as kuli-kuli, dankuwa, alewa etc. and engage in fishing activities [44]. As identified by the REA, Lade II is one of such non-electrified villages in Nigeria having a population of approximately 1200 people. Fig. 1 shows an aerial view of Lade II as retrieved from Google maps. In this paper, we assumed four (4) persons per household, consequently, 300 households were used in this work for a population of 1200 people.

**Fig. 2:**
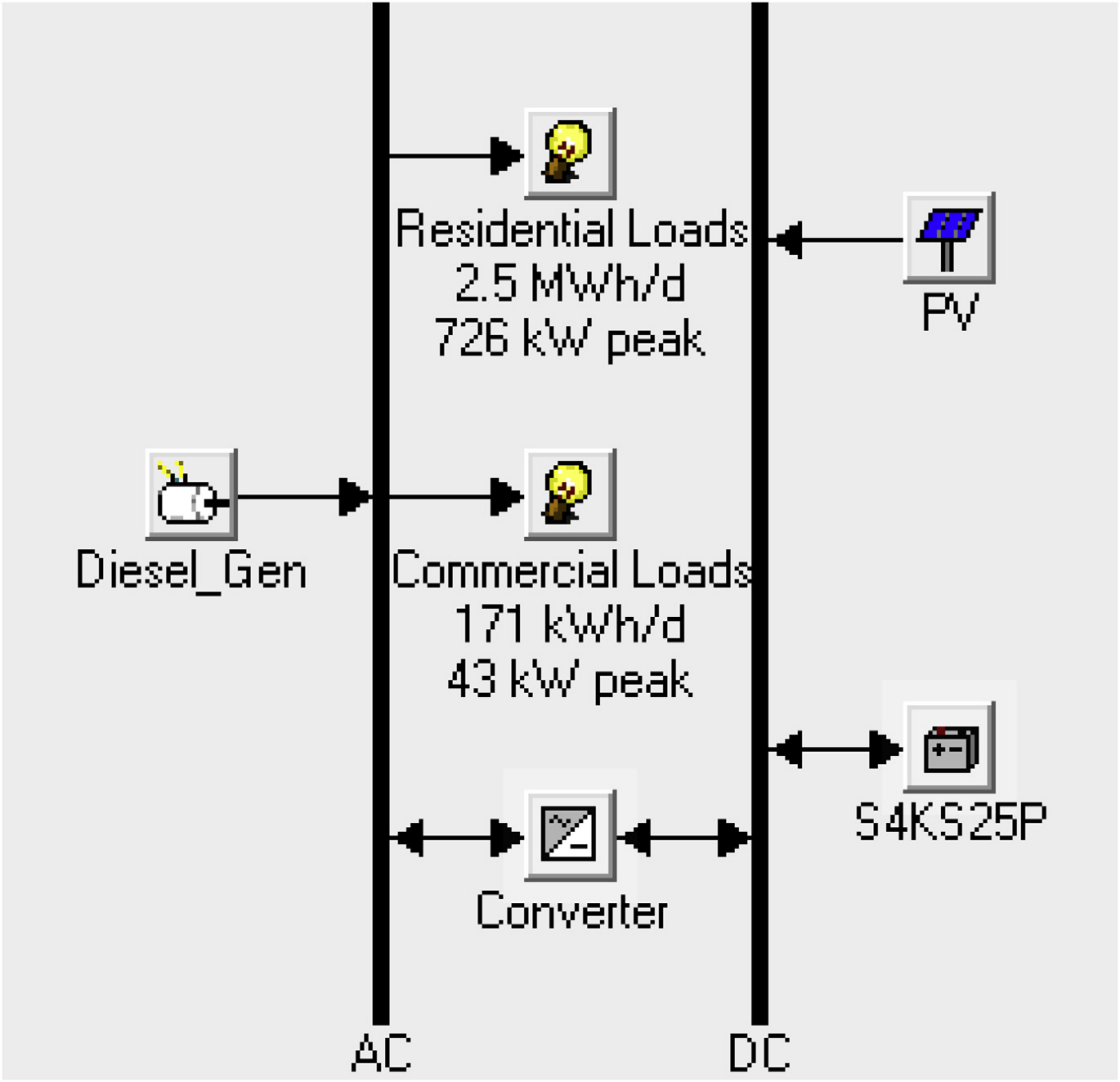
Schematic diagram showing hybrid energy design in HOMER.

The two categories of loads in the area under investigation are the residential and commercial loads. Three hundred (300) households were considered for the residential loads and thirty (30) small and medium scale enterprises (SME's) were considered for the commercial loads. Using the appliances power ratings as obtained from online retail stores in Nigeria such as Jumia [46] and other correspondence in [47], the load profile for a typical residential home was obtained as shown in Table 1 and then scaled up to ascertain the total real power, reactive power (using the power factor for each appliance), and the energy consumed by the residential & commercial loads. For this work, the same model of appliances was assumed for all residential and commercial load centers considered. Since the HOMER software requires an hourly power input for a whole year, a feature in the software that allows daily hourly power consumption to be entered alongside a random variability field comprising of a day-to-day random variability and a time-step-to-time-step value variability is used. This computes the hourly power consumption for a whole year thus making the synthetic data fed into the software more realistic. In this work, the day-to-day random variability value used was 15% and the time-step-to-time-step value used was 20% for both the residential and commercial loads. Furthermore, the per hour energy consumption of each appliance was fed into HOMER for simulation. And as seen from the schematic diagram in Fig. 2, the total energy consumed by the residential load was given as 2.5MWh/day with a peak load of 726kW.

**Table 1:** Load profile for Lade II, Pategi LGA, Kwara state.

S/N.	Appliances	Power Rating (W)	Qty.	Hrs. Used	Total Real Power Used (W)	PF	Total Reactive Power Used (VAR)	Energy Consumed (Wh)	Total (kWh)
**Residential Loads**									
1.	Television	41	1	7	41	0.8	30.750	287	
2.	Decoder	21	1	7	21	0.7	21.424	147	
3.	CFL's	18	5	12	90	0.56	133.151	1080	
4.	Fans	75	2	7	150	0.6	200.000	1050	
5.	Fridge	400	1	12	400	0.65	467.652	4800	
6.	Electric Iron	1000	1	1	1000	1	0.000	1000	
Daily total energy consumed per household:								8.364 kWh	
Daily total energy consumed for 300 households:								2509 kWh	**2509**
**Commercial Loads (Assume working hours of 9:00 am – 9:00 pm).**									
*SME 1 (Barbing Shops)*									
1.	CFL's	18	2	12	36	0.56	53.260	432	
2.	Fans	75	1	12	75	0.6	100.000	900	
3.	Television	41	1	12	41	0.8	30.750	492	
4.	Decoder	21	1	12	21	0.7	21.424	252	
5.	Electric Clipper	15	2	8	30	0.8	22.500	240	
Daily total energy consumed per shop:								2.316 kWh	
Daily total energy consumed for 10 shops:								23.16 kWh	
*SME 2 (Beauty Saloons)*									
1.	CFL's	18	2	12	36	0.56	53.260	432	
2.	Fans	75	1	12	75	0.6	100.000	900	
3.	Television	41	1	12	41	0.8	30.750	492	
4.	Decoder	21	1	12	21	0.7	21.424	252	
5.	Electric Hair Dryer	1500	1	4	1500	0.92	638.997	6000	
Daily total energy consumed per saloon:								8.076 kWh	
Daily total energy consumed for 10 saloons:								80.76 kWh	
*SME 3 (Small Restaurants, Mini-malls)*									
1.	CFL's	18	2	12	36	0.56	53.260	432	
2.	Fans	75	1	12	75	0.6	100.000	900	
3.	Fridge	400	1	12	400	0.65	467.652	4800	
Daily total energy consumed per space:								6.132 kWh	
Daily total energy consumed for 10 spaces:								61.32 kWh	
Daily total energy consumed by all Commercial Loads:								165.24kWh	**165.24**
Total energy consumed by Residential and Commercial Loads:									**2674.24**

**Fig. 3:**
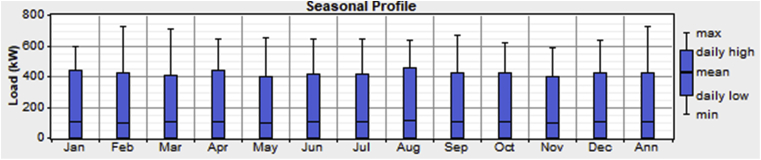
Seasonal load profile (residential loads).

**Fig. 4:**
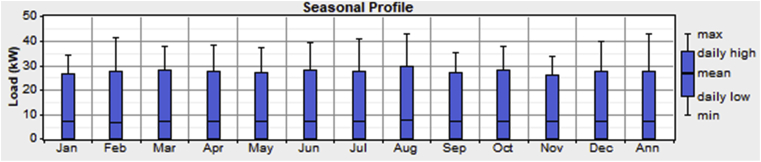
Seasonal load profile (commercial loads).

Table 1 also shows the profile of the commercial loads for the three different SME's considered in this work for all the outlets in the village. Work hours between 9:00 am and 9:00 pm were assumed. As seen from Table 1, SME 1, SME 2, and SME 3 has a total daily energy consumption of 23.16kWh, 80.76kWh & 61.32kWh respectively. The schematic shown in Fig. 2 shows a total energy consumption of 171kWh/day with a peak load of 43kW. The seasonal load profile for the residential and commercial load is as shown in Figs. 3 and 4.

It can be observed from Fig. 3 that the residential load consumption is highest for the months of February and March. This may be due to the high utilization of electric fans for cooling as higher temperatures and relatively low humidity are experienced within these months; hence more electricity is produced from the Solar PV's during this period. Fig. 4 shows the months of February and August as having the highest commercial load consumption period.

### 2.2 Solar and wind resource assessment

**Fig. 5:**
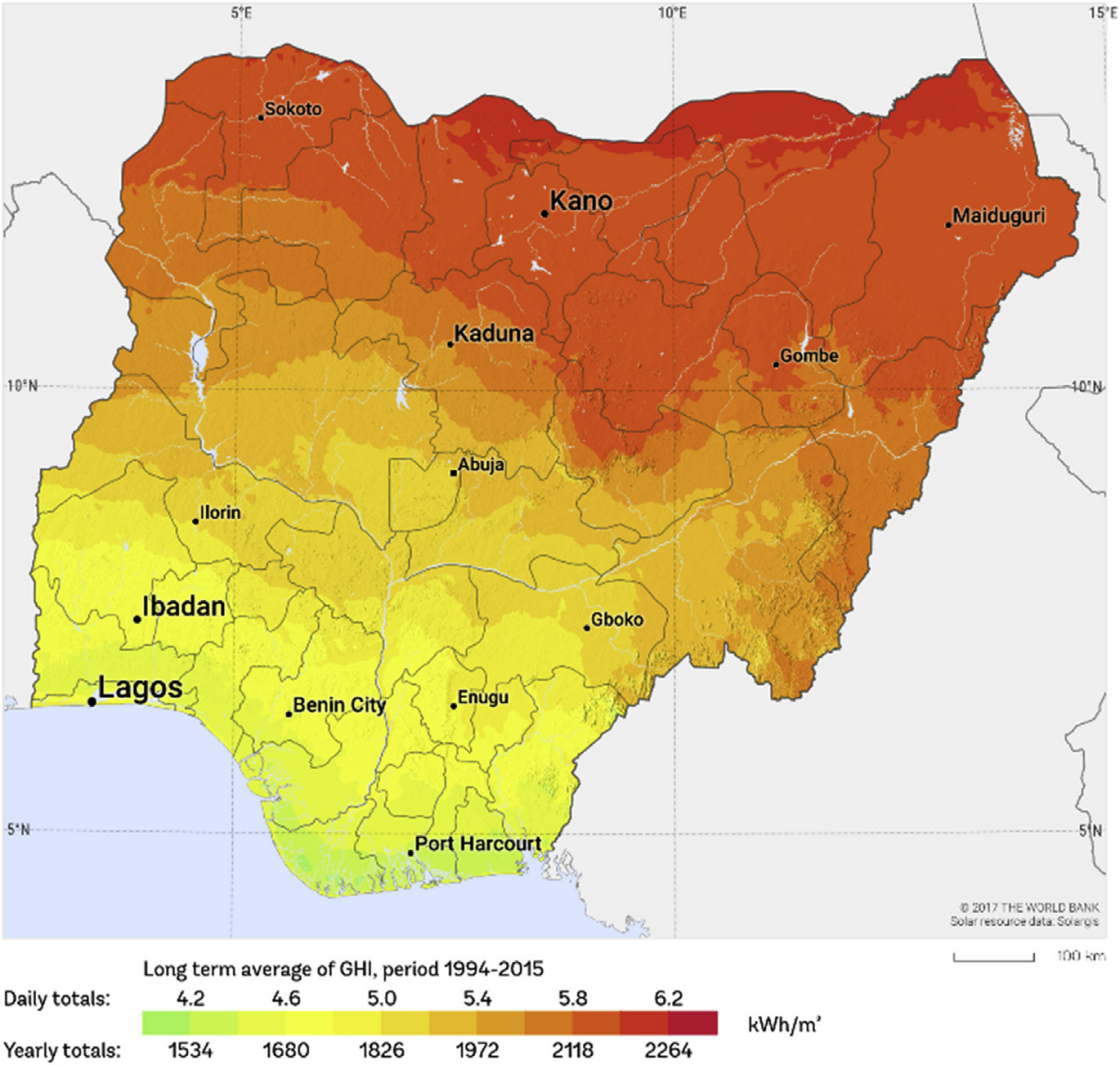
Global Horizontal Irradiation (GHI) map for Nigeria [49].

Nigeria is geographically located in the tropical region of the world and therefore experiences abundant solar insolation across the country even though some regions experiences better solar insolation than others. For example, as one transits to the north from the south, the solar insolation increases gradually. This is clearly depicted on the Global Horizontal Irradiation (GHI) map for Nigeria in Fig. 5. Fig. 5 is a solar resource data obtained from the Global Solar Atlas, owned by the World Bank Group and provided by Solargis [48].

The solar and wind resource for Lade II with the geographical co-ordinates 8.586453^o^N latitude & 5.526123^o^E longitude was obtained from the National Aeronautics and Space Administration (NASA's) power project website [50] having a 30-year meteorological data spanning between January 1984 & December 2013 and a 22-year additional monthly solar parameter and annual climatology data spanning between July 1983 and June 2005. Table 2 shows the monthly Insolation incident on a Horizontal surface (kWh/m^2^/day) and wind speed at 50m (m/s).

**Table 2:** Solar insolation and wind speed for lade II.

Month	Solar Insolation (kWh/m^2^/day)	Wind Speed (m/s)
January	5.74	3.21
February	5.91	3.43
March	6.01	3.85
April	5.78	4.26
May	5.43	3.93
June	4.92	3.85
July	4.44	4.14
August	4.26	4.11
September	4.55	3.27
October	5.12	2.96
November	5.72	2.67
December	5.67	2.92
**Average:**	**5.30**	**3.55**

The data in Table 2 shows a moderately high solar insolation for Lade II with an average of 5.30kWh/m^2^/day, hence harnessing solar energy through the deployment of Renewable Energy Technology (RET) would prove economically viable and feasible. Conversely, the wind speed in Lade II is quite poor with a yearly average of about 3.55 m/s at 50m above ground level. This speed is too low and as such wind turbines are not considered as one of the RET's to be used here as this is not economically viable. Furthermore, the wind turbines may not operate at their optimum capacities if deployed.

### 2.3 Hybrid system design

The hybrid system for Lade II comprises both solar and diesel energy sources with battery storage as a backup. The stochastic nature of the solar irradiation at the site necessitates the need for the diesel gen-sets to support base loads during periods when solar irradiation is low and battery capacity is at a minimum. We proceed to succinctly assess components used in the hybrid mini-grid design.

The solar PV was modeled into the HOMER software with a no-tracking capability, a derating factor of 80%, and a lifetime of 25 years since solar PV's requires very little maintenance and could hence last very long. The derating factor represents the variation between the rated and actual performance of the PV module because of factors such as ageing wiring losses, high temperature, shading, snow etc. Using the prevailing market prices of solar PV panels obtained from a popular retail store in Nigeria and a conversion rate of 1 USD ≈350 Naira, a capital cost of $664 per kW [51], a replacement cost of $580 was used as the prices of Solar PV's are expected to decline further in coming years. An O&M cost of $10/year was fed into HOMER. The solar PV output is obtained using Eq. (1) [52]:

(1) $$P_{pv}=Y_{pv}f_{pv}\left(\frac{G_{T}}{G_{T,STC}}\right)\left[1+\;\propto_{p}\left(T_{c}-\;T_{C,STC}\right)\right]$$

where $Y_{pv}$ is the rated capacity of the PV in kW, $f_{pv}$ is the PV derating factor, $G_{T}$ is the solar radiation incident on the PV array in kW/m^2^, $G_{T,STC}$ is the incident radiation at standard test conditions (1 kW/m^2^).

For the battery storage system, the Surrette 4KS25P batteries was selected from the already available battery catalog modeled into HOMER. The batteries used in this design were connected in three (3) strings producing a cumulative voltage of 12V. The capital cost per quantity of the battery was taken as $1221 [53], with its replacement cost taken as $1150 and O&M cost set at $10/year. The detailed technical specifications for the Surrette 4KS25P batteries used is shown in [54]. The battery storage capacity is given by Eq. (2) [55]:

(2) $$C_{wh}=\left(E_{L}\;\times \;AD\right)/\;\left(\eta_{inv}\times \;\eta_{Batt}\;\times DOD\right)$$

where $E_{L}$ is the average daily load energy (kWh/day), AD represents the number of days of battery autonomy, DOD is the depth of discharge of the battery while $\eta_{inv}$ and $\eta_{Batt}$ represent the inverter and battery efficiencies respectively.

The diesel generator used as a backup is modeled based on its fuel consumption (F_G_) pattern, which is proportional to its output power and given by Eq. (3) [55]:

(3) $$F_{G}=\;B_{G}\;\times \;P_{G-rated}+\;A_{G}\times \;P_{G-out}$$

where $B_{G}$ and $A_{G}$ represent the coefficient of the fuel consumption curve as specified by the designer (typically 1/kWh). $P_{G-rated}$ is the nominal power of the diesel generator and $P_{G-out}$ is the output power of the generator. Using the prevailing market prices of Perkins sound-proof diesel generators obtained from an online retail store in Nigeria and a conversion rate of 1 USD ≈350 Naira, a cumulative capital cost of $48,286 [56, 57, 58] comprising individual costs of 200kVA, 100kVA, and 50kVA generators respectively. Replacement cost was set at $42,000 and an overall O&M cost of $133/hr was utilized in the mini-grid design to cater for the costs of repairs or replacements of component parts.

Lastly, a bi-directional converter was used in the design and since the system followed a load following strategy (LF), the diesel generator produced only enough power to serve the loads and did not charge the batteries. The capital cost per kW for the converter was taken as $840 [59], replacement cost at $700 and the O&M cost at $8/year.

### 2.4 Hybrid system optimization model and economic evaluation

In achieving the optimal hybrid solution, HOMER accesses the decision variables used as represented in Fig. 2, and optimizes based on an objective function which considers the total life cycle cost of the hybrid system. The decision variables utilized in this work are based on the available system resources and the load demand. The system assets used incorporates the solar PV size, size of the diesel generator, number of batteries and the converter size. The objective function is thus given by Eq. (4) [60]:

(4) $$\text{Minimize:}\;C_{ann}=\;\underset{n}{\sum }\left(C_{ann,cap}+C_{ann,rep}\;+C_{ann,O\&M}\right)$$

where n is the number of units of each system component used, which comprises the solar PV panel, diesel generator, battery and converter. $C_{ann,cap}$ is the annualized capital, $C_{ann,rep}$ is the replacement cost and $C_{ann,O\&M}$ is the operating and maintenance cost of each system component. Eq. (4) can also be re-written as Eq. (5) [60]:

(5) $$C_{ann\left(n\right)}=Q\;\times \left\{\left[C_{ann,cap}+\;C_{ann,rep}\;\times \;K_{n}\;\left(i,\;L_{n},\;y_{n}\right)\right]\times CRF\;\left(i,\;N\right)+\;C_{ann,O\&M}\right\}\;$$

where Q is the quantity of components, CRF is the capital recovery factor given by Eq. (6) [20], K is the single payment present worth factor given by Eq. (7) [60].

(6) $$CRF\;\left(i,\;N\right)=\;\frac{{i\left(1+i\right)}^{N}}{{\left(1+i\right)}^{N}-1}$$

where N and i are the number of years and annual real interest rate respectively.

(7) $$K_{n}\;\left(i,\;L_{n,\;}y_{n}\right)=\;\overset{y_{n}}{\underset{x=1}{\sum }}\frac{1}{{\left(i+1\right)}^{x\;\times L_{n}}}$$

where L and y are the useful lifetime and the number of component replacement during the project lifetime respectively.

For each search space in the decision variable that HOMER uses in its optimization procedure, the objective function is minimized subject to set imperatives, which incorporates: the energy balance constraints, the battery charging and discharging constraints, the solar PV constraints, and the diesel generator's technical constraints [60].

HOMER represents the life cycle cost of its energy solutions based on the total net present cost (NPC) obtained for each solution, with the optimal solution being the system combination having the least NPC. The total net present cost comprises of the initial capital cost, replacement cost, fuel costs as well as the annual operating and maintenance costs. The NPC is expressed as shown in Eq. (8) [20].

(8) $$C_{npc,\;tot}=\;\frac{C_{ann,\;tot}}{CRF\left(i,\;P_{lifetime}\right)}$$

where $C_{ann,\;tot}$ is the total annualized cost ($/year), i is the annual real interest rate (%), $P_{lifetime}$ is the project lifetime (years), and CRF represents the capital recovery factor as defined earlier in Eq. (6). The levelized cost of energy (LCOE), which is the tariff exerted on consumers for electricity purchased per kWh is determined using Eq. (9).

(9) $$COE=\;\frac{C_{ann,\;tot}}{E_{ann,\;load}}$$

where $C_{ann,\;tot}$ is the total annualized cost and $E_{ann,\;load}$ is the total annual load served by the system in kWh [20].

### 2.5 Reliability evaluation

There is the need to determine if a generation expansion schedule (in our case the hybrid energy design for Lade II) satisfies the desired level of reliability defined by the following three reliability indices used in this work: Loss of Load Probability (LOLP), Loss of Load Expectation (LOLE) and Expected Load Loss (ELL). To carry out these reliability evaluations, the performance characteristics of each generating source used in the hybrid energy system design need to be known. In this work, we limit this to only the Forced Outage Rate (F.O.R) or failure rate of each of the generating sources.

The reliability model of a generating plant can be represented by a table called the Capacity Outage Probability Table (COPT) according to the available generation capacity and corresponding cumulative probabilities. The elements in a COPT can be expressed as shown in Eq. (10) [40].

(10) (C_i_, F (C_i_)) = (C_i_, Prob (P (k, λ) ≤ C_i_) i ε 1, 2… N

where C_i_ is the average capacity in ith interval, N is the number of states, λ represents the forced outage rate and k represents the probability feature for any RET obtained from any of its parameters. For a wind turbine generator, this is the wind speed and for a solar PV array, this could be the solar insolation or clearness index.

A COPT is developed where the various generating plant sizes with their commensurate failure rates are used to compute the reliability indices required in this work. The F.O.R's, also referred to as unit availability/failure rate is given by the formula shown in Eq. (11) [61]:

(11) $$F.O.R=\;\frac{\lambda }{\lambda +\;\mu }$$

where λ represents the expected failure rate and μ represents the expected repair time.

As indicated in [62], the F.O.R's for a Solar PV, Diesel generator and Battery are 0.03, 0.06 and 0.04 respectively. Table 3 gives shows the F.O.R's and lifetime years of each generating source used in the system design. The lifetime years are as specified and simulated in the HOMER software.

**Table 3:** Generating Sources lifetime and failure rates.

Generators	Forced Outage Rates (F.O.R)	Lifetime
Solar PV	0.03	25 years
Diesel Generator	0.06	15,000 operating hours
Battery	0.04	Minimum of 10 years.

### 2.6 Reliability indices (LOLP, LOLE, ELL)

Loss of load probability is the probability of the system's daily peak or hourly demand exceeding the available generating capacity during a given period [63]. In other words, it represents the expected number of days in which the peak load will exceed the available installed capacity. The LOLP reliability index is calculated using Eq. (12) [62]:

(12) $$LOLP=\overset{n}{\underset{i=1}{\sum }}P\;\left(C_{i}\right)\;P\left(L_{i}>C_{i}\right)$$

Where $P\;\left(C_{i}\right)$ is the probability of a loss in capacity and $P\left(L_{i}>C_{i}\right)$ is the duration in percentage of the loss of capacity. The LOLE is a probabilistic measure which indicates the risk at which the generation capacity fails to meet the demand [61]. It is generally defined as the expected number of days per year for which the available generation capacity is insufficient to serve the daily peak demand of the load points. The LOLE is computed using Eq. (13) [62]:

(13) $$LOLE=\overset{n}{\underset{k=1}{\sum }}P_{k}t_{k}$$

Where $P_{k}$ is the individual probability of capacity in outage and $t_{k}$ is the duration of loss of power supply in days. Whereas the LOLP and LOLE indicates the probabilistic measure of load lost in days (or hours) per year, it fails to provide a quantifiable value of just how much that load loss is. Given the hybrid system's total installed capacity and peak load demand of the village considered, the cumulative product of the load lost in MW and the probability of load lost produces the total expected load loss of the hybrid system. This is defined by Eq. (14), and can be obtained from the COPT generated for the hybrid system [40].

(14) $$ELL=\overset{n}{\underset{i=1}{\sum }}Load\;Lost\;\left(MW\right)\;\times Probability\;of\;Load\;Lost\;$$

In summary, the reliability indices assessment for the HOMER designed hybrid mini-grid followed the steps as outlined:

1) Determination of the total installed capacity and peak load from the hybrid system results produced by the HOMER software.
2) Development of Individual COPT for each generating source.
3) Combination of the Individual COPT and Re-ordering of the combined COPT.
4) Computing of the LOLP, LOLE & ELL based on Eqs. (12), (13), and (14).

## 3 Results & discussion

### 3.1 Optimization result

Sequel to the Hybrid system modeling which utilized the energy components and prices, load demand data, and meteorological data, HOMER performed simulations on the model which took several hours due to the complexity of the designed system and the number of sensitivity variables considered. During these simulations, HOMER objectively classified each system combination based on the net present cost, operating cost per year, initial capital, cost of electricity, renewable fraction, and gasoline consumption. It then selected the system combinations which had the least cost of electricity and net present cost as its optimal solution.

As seen from Table 4, the optimization results for Lade II reveals an optimal result comprising a 1,500kW solar PV, 350kW diesel generator, 1200 units of batteries with a usable nominal capacity of 5,472kWh, and a 600kW converter. The cost summary for the system is shown in Table 5. The optimal system showed a load following dispatch strategy (LF) where the diesel generators only provided enough power to serve the load at any particular point in time and do not charge the batteries. The batteries were solely being charged by the renewable energy source i.e. the solar PV's. The diesel generators would operate for 212 hours per year with an electricity production of 45,958kWh per year and total fuel consumption of 18,425 liters per year. Contrasting the optimal hybrid system architecture with the diesel-only system seen in Table 4, even though the initial capital required for the diesel-only system was far lesser, there were very significant increases in its operating costs, total NPC, COE, diesel fuel consumption and the number of hours required for the diesel generator's operation. Consequently, with the hybrid system, there was a proportionate reduction of approximately 97% in the emissions outputs when compared with the diesel-only system as seen in Table 6.

**Table 4:** Optimization results as obtained from HOMER.

System Components Sizing					Initial Capital ($)	Operating Cost ($/yr.)	Total NPC ($)	COE ($/kWh)	RF	Diesel Fuel (Liters)	Diesel Gen (Hrs.)
S/N	Solar PV (kW)	Diesel Gen (kW)	Batteries (Units)	Converter (kW)							
**1.**	**1500**	**350**	**1200**	**600**	**3,013,486**	**148,296**	**4,909,206**	**0.396**	**0.98**	**18,425**	**212**
2.	2000	0	1650	600	3,846,650	143,619	5,682,588	0.459	1	0	0
3.	0	350	750	200	1,132,036	791,462	11,249,571	0.908	0	404,783	3,694
4.	1600	450	0	300	1,376,482	975,569	13,847,526	1.118	0.78	334,672	4,255
**5.**	**0**	**500**	**0**	**0**	**68,980**	**1,782,979**	**22,861,434**	**1.846**	**0**	**614,924**	**7,299**

**Table 5:** Cost summary for optimal hybrid energy configuration.

Component	Capital ($)	Replacement ($)	O & M($)	Fuel ($)	Salvage ($)	Total ($)
PV	996,000	271,270	191,750	0	-152,032	1,306,989
Diesel Gen	48,286	0	360,440	141,323	-6,328	543,721
Surrette 4kS25P	1,465,200	1,026,649	153,400	0	-294,744	2,350,506
Converter	504,000	175,251	61,360	0	-32,620	707,992
**System**	3,013,486	1,473,171	766,951	141,323	-485,724	4,909,207

**Table 6:** Comparison between environmental pollutants of the hybrid optimal system and diesel-only system.

Pollutants	Optimal Hybrid Emissions (kg/year)	Diesel-Only Emissions (kg/year)
Carbon dioxide	48,520	1,619,296
Carbon monoxide	120	3,997
Unburned Hydrocarbons	13.3	443
Particulate matter	9.03	301
Sulfur dioxide	97.4	3,252
Nitrogen Oxides	1,069	35,666

**Fig. 6:**
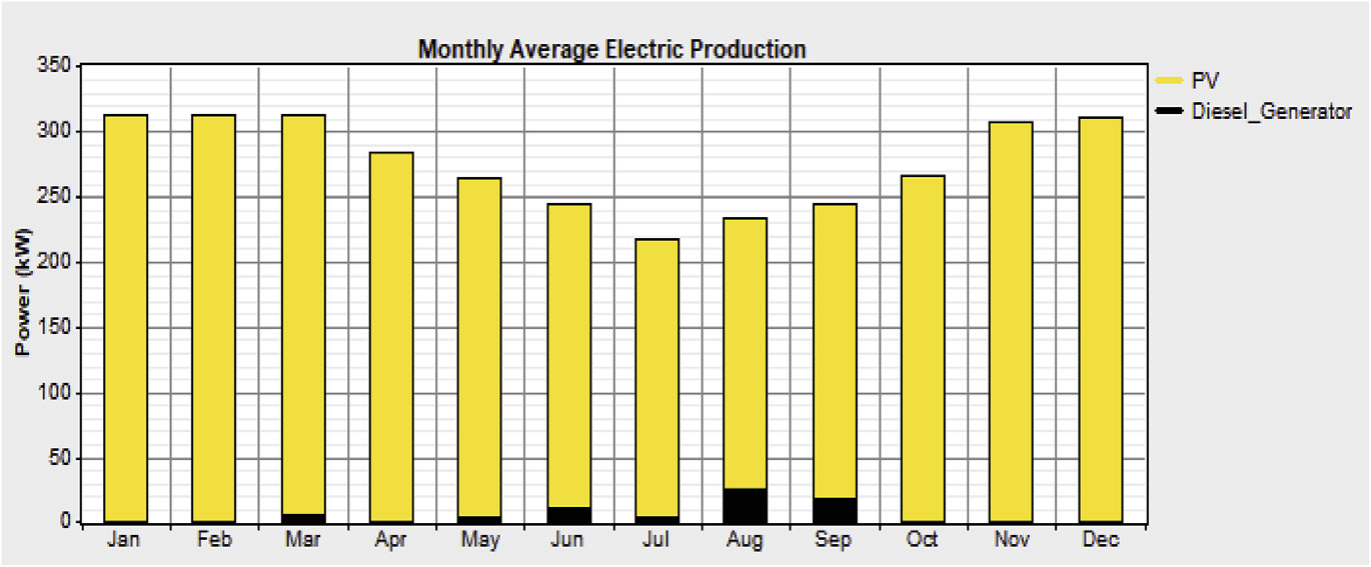
Average monthly electricity production from Solar PV's and diesel generators.

The total electricity produced from the diesel generators constitutes only 2% as compared to that from the Solar PV's which contributes 98% of the total, thereby making the renewable fraction (RF) of the hybrid system design 0.979. Fig. 6 shows the average monthly electricity production from the Solar PV's and diesel generators.

### 3.2 Sensitivity analysis

Some sensitivity variables were fed into HOMER to determine the optimal system combination and commensurate techno-economic analysis for such systems. In conducting the sensitivity analysis in this work, a variation in the solar insolation, fluctuations in diesel prices, and variations in both the capital and replacement costs of solar PV's was conducted.

### 3.3 Sensitivity analysis results with variation in solar insolation

During the hybrid system design in HOMER, the average solar insolation was varied between 4.9kWh/m^2^/day and 5.3kWh/m^2^/day. As observed in Table 7, with all other system parameters unchanged, when the average solar insolation was 4.9kWh/m^2^/day, the size of solar PV needed was found to be 1600kW, which is 100kW more than when the average solar insolation was 5.3kWh/m^2^/day. Consequently, the initial capital cost, operating cost, total NPC and COE was seen to increase by approximately 2.2%, 1.1%, 1.8%, and 1.8% respectively as compared to when the solar insolation was 5.3kWh/m^2^/day.

**Table 7:** Sensitivity analysis results.

Sensitivity Variables.	PV (kW)	Diesel Gen (kW)	S4kS25P (No of Units)	Conv. (kW)	Initial Capital ($)	Operating Cost ($/yr.)	Total NPC ($)	COE ($/kWh)	RF	Diesel (L)

**Solar insolation variations**										
5.3kWh/m^2^/day	1500	350	1200	600	3,013,486	148,296	4,909,206	0.396	0.98	18,425
4.9kWh/m^2^/day	1600	350	1200	600	3,079,886	149,959	4,996,868	0.403	0.98	18,271
**Diesel price variations at avg. solar insolation of 5.3kWh/m**^**2**^**/day**										
$0.4/L	1500	350	1200	600	3,013,486	148,822	4,839,227	0.391	0.98	17,981
$0.6/L	1500	350	1200	600	3,013,486	148,296	4,909,206	0.396	0.98	18,425
$0.7/L	1500	350	1200	600	3,013,486	150,139	4,932,760	0.398	0.98	18,425
**Diesel price variations at avg. solar insolation of 4.9kWh/m**^**2**^**/day**										
$0.4/L	1600	350	1200	600	3,079,886	145,802	4,943,722	0.399	0.98	18,355
$0.6/L	1600	350	1200	600	3,079,886	149,959	4,996,868	0.403	0.98	18,271
$0.7/L	1600	350	1200	600	3,079,886	151,786	5,020,224	0.405	0.98	18,271
**Solar PV capital and replacement cost multiplier variations at avg. solar insolation of 5.3kWh/m**^**2**^**/day and $0.4/L diesel price**										
1 ($664/kW)	1500	350	1200	600	3,013,486	142,822	4,839,227	0.391	0.98	17,981
0.5 ($332/kW)	2000	350	1050	600	2,498,336	139,298	4,279,037	0.345	0.99	15,241
**Solar PV capital and replacement cost multiplier variations at avg. solar insolation of 5.3kWh/m**^**2**^**/day and $0.6/L diesel price**										
1 ($664/kW)	1500	350	1200	600	3,013,486	148,296	4,909,206	0.396	0.98	18,425
0.5 ($332/kW)	2000	350	1050	600	2,498,336	143,486	4,332,566	0.350	0.99	15,349
**Solar PV capital and replacement cost multiplier variations at avg. solar insolation of 5.3kWh/m**^**2**^**/day and $0.7/L diesel price**										
1 ($664/kW)	1500	350	1200	600	3,013,486	150,139	4,932,760	0.398	0.98	18,425
0.5 ($332/kW)	2000	350	1050	600	2,498,336	145,021	4,352,188	0.351	0.99	15,349
**Solar PV capital and replacement cost multiplier variations at avg. solar insolation of 4.9kWh/m**^**2**^**/day and $0.4/L diesel price**										
1 ($664/kW)	1600	350	1200	600	3,079,886	145,802	4,943,722	0.399	0.98	18,355
0.5 ($332/kW)	1700	350	1200	600	2,581,886	140,678	4,380,227	0.354	0.98	15,255
**Solar PV capital and replacement cost multiplier variations at avg. solar insolation of 4.9kWh/m**^**2**^**/day and $0.6/L diesel price**										
1 ($664/kW)	1600	350	1200	600	3,079,886	149,959	4,996,868	0.403	0.98	18,271
0.5 ($332/kW)	1700	350	1200	600	2,581,886	146,027	4,448,602	0.359	0.98	15,952
**Solar PV capital and replacement cost multiplier variations at avg. solar insolation of 4.9kWh/m**^**2**^**/day and $0.7/L diesel price**										
1 ($664/kW)	1600	350	1200	600	3,079,886	151,786	5,020,224	0.405	0.98	18,271
0.5 ($332/kW)	1700	350	1200	600	2,581,886	147,622	4,468,993	0.361	0.98	15,952

### 3.4 Sensitivity analysis results with variation in diesel price

The global oil and gas sector experiences fluctuations in the prices of processed crude oil products, among the several causes of these price fluctuations are governmental policies and complexities arising from the extraction and processing of crude oil into products such as diesel fuel. Hence, a sensitivity analysis of fluctuations in diesel prices becomes necessary. In this work, when the prices of diesel were varied between $0.4/L, $0.6/L and $0.7/L at 5.3kWh/m^2^/day, it was observed that prices of diesel influenced the overall NPC and COE as seen in Table 7. The higher the cost of diesel fuel, the higher the cost of electricity became and the same trend was observed when the average solar insolation was set at 4.9kWh/m^2^/day. At an average solar insolation of 4.9kWh/m^2^/day, due to the change in the component size of the solar PV and the cumulative effects of fluctuations in diesel prices, the total NPC observed at $0.4/L, $0.6/L and $0.7/L increased in proportion by 2.2%, 1.8% and 1.8% respectively as compared to having the average solar insolation at 5.3kWh/m^2^/day. Likewise, the cost of electricity (COE) observed at $0.4/L, $0.6/L and $0.7/L increased by 2%, 1.8%, and 1.8% respectively.

### 3.5 Sensitivity analysis with variations in solar PV capital and replacement costs

According to Swanson's law, the more a product is being manufactured, the cheaper it gets. As a result, there has been a rapid decline in the prices of solar PV's technologies in recent times. Therefore, this necessitates a sensitivity analysis of the variations in the costs of solar PV's. In this work, the solar PV capital cost and replacement costs were linked together during the system configuration in HOMER, hence a change in the capital cost automatically produces a commensurate change in the replacement costs. The solar PV multiplier used was varied between 1 and 0.5 as indicated in Table 7, this signifies a variation in the capital cost between $664 per kW and $332 per kW.

At an average solar insolation of 5.3kWh/m^2^/day, and diesel price fluctuations between $0.4/L and $0.7/L, varying the capital cost of solar PV between $664/kW and $332/kW, the total NPC, COE and diesel fuel consumption in liters was observed to reduce by 12%, 12%, and 15% respectively at a diesel price of $0.4/L. However, when the diesel prices were $0.6/L and $0.7/L, the reduction in total NPC, COE and diesel fuel consumption in liters observed was 12%, 12%, and 17% respectively. At an average of 4.9kWh/m^2^/day and diesel price fluctuations between $0.4/L and $0.7/L, when solar PV capital cost is varied from $664/kW to $332/kW, the proportionate reduction in the total NPC, COE and diesel fuel consumption was 11%, 11% and 17% respectively when diesel fuel cost was $0.4/L. However, when the prices of diesel were $0.6/L and $0.7/L, the reduction in total NPC, COE and diesel fuel consumption obtained was 11%, 11%, and 13% respectively.

### 3.6 Reliability results

The optimal simulation result from HOMER was tested for its reliability. The optimized solution as indicated in Table 4 showed three (3) generating sources i.e. solar PV, diesel generator and battery storage system (System Architecture). The COPT for each generating source is grouped and is as shown in Tables 8, 9, and 10. Group 1 comprised of 2 no's of 0.75MW solar PV giving a total of 1.5MW. Group 2 comprised of 2 no's 0.175MW Diesel generators giving a total of 0.350MW and Group 3 comprised of 3 no's 1.824MWh Surrette batteries giving a total usable nominal capacity of 5.472MWh.

**Table 8:** COPT for Group 1 (2 no's 0.75MW solar PV).

Units Out	Capacity Out (MW)	Capacity In (MW)	Binomial Expansion (p + q)^2^	Probability
0	0	1.5	p_1_^2^	(0.97)^2^ = 0.9409
1	0.75	0.75	2p_1_q_1_	2 (0.97) (0.03) = 0.0582
2	1.5	0	q_1_^2^	(0.03)^2^ = 0.0009

Where q_1_ represents the Failure rate or unit unavailability of the Solar PV given by 0.03 or 3%. p_1_ represents the unit availability given by 0.97 or 97%.

**Table 9:** COPT for Group 2 (2 no's 0.175MW Diesel Generator).

Units Out	Capacity Out (MW)	Capacity In (MW)	Binomial Expansion (p + q)^2^	Probability
0	0	0.35	p_2_^2^	(0.94)^2^ = 0.8836
1	0.175	0.175	2p_2_q_2_	2 (0.94) (0.06) = 0.1128
2	0.35	0	q_2_^2^	(0.06)^2^ = 0.0036

Where q_2_ represents the Failure rate or unit unavailability of the Diesel generator given by 0.06 or 6%. p_2_ represents the unit availability given by 0.94 or 94%.

**Table 10:** COPT for Group 3 (3 no's 1.824MWh Surrette 4KS25P batteries).

Units Out	Capacity Out (MW)	Capacity In (MW)	Binomial Expansion (p + q)^2^	Probability
0	0	5.472	p_3_^3^	(0.96)^3^ = 0.884736
1	1.824	3.648	3p_3_^2^q_3_^1^	3 (0.96)^2^ (0.04) = 0.110592
2	3.648	1.824	3p_3_^1^q_3_^2^	3 (0.96) (0.04)^2^ = 0.004608
3	5.472	0	q_3_^3^	(0.04)^3^ = 0.000064

Where q_3_ represents the Failure rate or unit unavailability of the battery given by 0.04 or 4%. p_3_ represents the unit availability given by 0.96 or 96%.

A summary reliability results table is as shown in Table 11. Using the COPT, the performance of the simulation result from HOMER was validated.

**Table 11:** Summary reliability result table.

Indicators	System Architecture
Peak load	769kW
Load loss (zero diesel gen, zero solar PV & battery)	769kW
Load loss (one diesel gen, zero solar PV & battery)	594kW
Load loss (two diesel gen, zero solar PV & battery)	419kW
Loss of load probability (LOLP)	0.00000576%
Loss of load expectation (LOLE)	0.000021024 day/year or 0.00050457hrs/year.
Total expected load loss (ELL)	0.025344Watts

However, comparing the reliability results obtained in this work with that obtained from a similar work performed by authors in [43], Table 12 presents the system architecture used in [43] alongside the LOLP and LOLE obtained. The system components sizing for the work in [43] was optimized using the Fmincon optimization tool in Matlab while HOMER was used for the optimal system component sizing used in this work.

**Table 12:** Comparison of reliability results obtained with previous work.

S/N	System Architecture	System component sizes in [43]	System component sizing in current research.
1.	Diesel Generator	48kW	350kW
2.	Solar PV's	300W	1500kW
3.	Wind Turbine	500W	Null
4.	Energy Storage System (ESS)	84Ah or 4kWh	5,472kWh
Reliability Indicators.			
1.	Loss of Load Probability (LOLP)	2.81 × 10^−10^	5.76 × 10^−8^
2.	Loss of Load Expectation (LOLE)	2.46 × 10^−6^ hr./year	5.0457 × 10^−4^ hr./year

In comparing the system architectures in Table 12, one key point observed was the hybrid combination of the RERs in [43] which comprised a solar PV, wind turbine, diesel generator and energy storage systems while that obtained in this work comprised of solar PV, diesel generator and energy storage systems. Hence, the loss of load probability and loss of load expectation observed in [43] was lesser than that obtained in this work.

## 4 Conclusion

This work performed the design and techno-economic analysis of a hybrid mini-grid system and also validated the reliability of adopting the simulation results obtained from the HOMER software for a typical rural community in Nigeria. As seen from the work, for a residential load demand of 2.5MWh/day and a commercial load demand of 165kWh/day, the optimal system results from HOMER comprised a 1500kW solar PV, 350kW diesel generator and 1200 units of deep cycle batteries having a net present cost, operating cost and cost per kWh of electricity of $4,909,206, $148,296 per year and $0.396/kWh respectively.

The sensitivity analysis results from varying the solar insolation, diesel fuel prices and solar capital/replacement costs showed an increase in the initial capital, operating cost, total NPC and COE by approximately 2.2%, 1.1%, 1.8% and 1.8% respectively when the average solar insolation dropped from 5.3kWh/m^2^/day to 4.9kWh/m^2^/day. Also, when the prices of diesel fuel were varied at each of the two different average solar insolation, the total NPC and COE observed at $0.4/L, $0.6/L and $0.7/L increased in proportion by approximately 2%, 1.8%, and 1.8% respectively. The sensitivity analysis results for the variations in the solar PV prices also recorded drastic reductions in diesel fuel consumption, total NPC and COE when the capital costs of solar PV's fell from $664/kW to $332/kW.

This work also utilized the capacity outage probability table (COPT) in validating the reliability of the optimal system combination results obtained from the HOMER software. Using the Forced outage rate of the system components, the values of the LOLP, LOLE and ELL obtained were 5.76 × 10^−8^, 5.0457 × 10^−4^ hrs./yr., and 0.025344Watt respectively. These results were compared with similar results obtained from a different hybrid system architecture comprising of a solar PV, wind turbine, energy storage systems and diesel generators having lower values of 2.81 × 10^−10^ and 2.46 × 10^−6^ hr./year respectively for its LOLP and LOLE. The difference being the system architecture used in this work which comprises of a solar PV, battery energy storage system and diesel generators only. A future work plan could consider the overall costs of the total expected load loss on the mini-grid utility and its effect on the sustainability of the mini-grid in the long run.

The reliability indicators obtained in this work being very small thus validates the optimal HMS solution as produced by HOMER and would offer quality energy supply to the rural community.

## Declarations

### Author contribution statement

Esan Ayodele Benjamin: Conceived and designed the experiments; Wrote the paper.

Agbetuyi Ayoade Felix: Performed the experiments.

Oghorada Oghenevogaga: Analyzed and interpreted the data.

Ogbeide Kingsley, Ayokunle Awelewa & Afolabi Esan: Contributed reagents, materials, analysis tools or data.

### Funding statement

This work was supported by Landmark University.

### Competing interest statement

The authors declare no conflict of interest.

### Additional information

No additional information is available for this paper.
